# Structural Stabilization of Tissue for Embryo Phenotyping Using Micro-CT with Iodine Staining

**DOI:** 10.1371/journal.pone.0084321

**Published:** 2013-12-30

**Authors:** Michael D. Wong, Shoshana Spring, R. Mark Henkelman

**Affiliations:** 1 Mouse Imaging Centre, Hospital for Sick Children, Toronto, Ontario, Canada; 2 Department of Medical Biophysics, University of Toronto, Toronto, Ontario, Canada; Osaka University Graduate School of Medicine, Japan

## Abstract

The International Mouse Phenotyping Consortium has been established to conduct large-scale phenotyping of the approximately 23,000 single-gene knockout mice generated by the International Knockout Mouse Consortium to investigate the role of each gene in the mouse genome. Of the generated mouse lines, 30% are predicted to be embryonic lethal, requiring the implementation of imaging techniques and analysis tools specific to late gestation mouse embryo phenotyping. A well-adopted technique combines the use of iodinated contrast solutions and micro-computed tomography imaging. This simple iodine immersion technique provides superior soft-tissue contrast enhancement, however, the hypertonic nature of iodine promotes dehydration causing moderate to severe tissue deformation. Here, we combine the stabilizing properties of a hydrogel mesh with the enhanced contrast properties of iodine. The protocol promotes cross linking of tissue through formaldehyde fixation and the linking of hydrogel monomers to biomolecules. As a result, the hydrogel supports tissue structure and preserves its conformation taking advantage of iodine-enhanced soft tissue contrast to produce high quality mouse embryo images with minimal tissue distortion. Hydrogel stabilization substantially reduces intersample anatomical variation of mature mouse embryos subjected to iodine preparation protocols. A 20% and 50% reduction in intersample variation of normalized brain and lung volume is achieved through hydrogel stabilization, as well as a 20% reduction in variation in overall embryo anatomy as measured through image registration methods. This increases the sensitivity of computer automated analysis to reveal significant anatomical differences between mutant and wild-type mice.

## Introduction

With the completion of the mouse genome sequence [1], the goal of the International Knockout Mouse Consortium (IKMC) is to generate mice with gene knockouts for each of the ∼23,000 genes in the genome in an attempt to investigate the relationship between genotype and expressed phenotype [2]. As this international effort is nearing conclusion, the International Mouse Phenotyping Consortium (IMPC, http://www.mousephenotype.org) was established in 2010 to conduct large-scale primary phenotyping required to determine the function of each gene. Along with adult mouse studies, the IMPC has developed a specific pipeline to investigate embryonic phenotypes. Mouse embryos are used to investigate the genetic basis of development and congenital abnormalities but are also critical in examining the 30% of single-gene knockout mice produced by the IKMC that will be embryonic lethal. The planned primary screen to investigate embryonic lethal knockout lines is based on three-dimensional (3D) imaging and statistical analysis of morphometric phenotypes [3].

**Figure 1:**
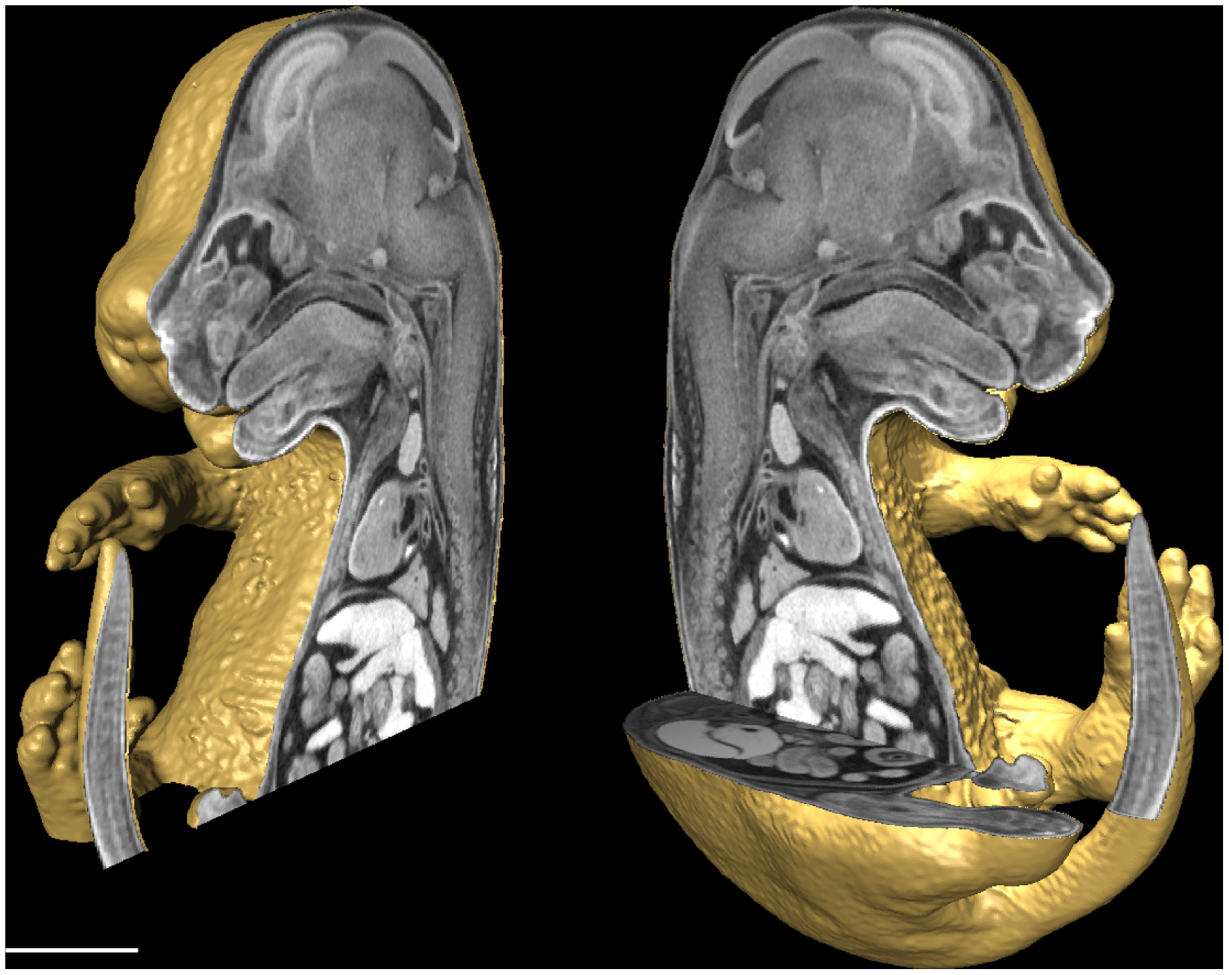
Micro-CT 3D image of a Lugol stained E15.5 mouse embryo allows for whole embryo coverage of mouse embryo anatomy. Micro-CT generates a 3D data set of mouse embryo morphology that can be digitally sectioned at any desired orientation. This wealth of digital information can be processed through computer automated analysis to localize structural differences in gene knockout mice. Scale bar, 2 mm.

Micro-computed tomography (micro-CT), an imaging modality based on the attenuation of X-rays, is the primary option for 3D imaging of late gestation mouse embryos in the IMPC pipeline. To increase the X-ray contrast of soft tissues, researchers have recently turned to iodinated contrast media to visualize the soft tissue of mouse embryos with great success [4], [5] as demonstrated by Fig. 1. In addition, we have previously presented that micro-CT images of mouse embryos stained with 0.025 N potassium tri-iodide (Lugol solution) are amenable to computer automated organ volume calculations, furthering the usefulness of micro-CT as a high-throughput phenotyping method [6]. Although the immersion staining technique is simple, the iodine solution potency and immersion time must be carefully balanced to provide appropriate contrast and to minimize tissue shrinkage and deformation that would complicate intersample statistical comparisons [7]. Despite optimization, some degree of tissue shrinkage appears unavoidable, which is particularly evident in tissues that have a higher proportion of water such as the brain and lung [6].

A new method for 3D tissue preparation, termed CLARITY, uses a hydrogel mesh to physically support organ tissues, like mouse brains, before transforming them into optically transparent structures using electrophoresis [8]. The resulting tissue is permeable to macromolecules used for specific staining and is transparent, allowing for visual access to large networks of neurons, protein complexes and nucleic acids. Here we utilize the tissue stabilization portion of the CLARITY protocol to minimize the tissue shrinkage caused by iodine staining. This protocol promotes cross linking of tissue through formaldehyde fixation and the linking of hydrogel monomers to biomolecules [8]. As a result, the hydrogel supports tissue structure and preserves its conformation, minimizing sample shrinkage and deformation inherent to the iodine staining protocol. This results in high quality 3D micro-CT mouse embryo images with remarkable soft tissue contrast and only minimal tissue distortion. We’ve designated this method STABILITY.

## Materials and Methods

### Sample Preparation

Plugged (natural matings) C57Bl/6 mice were sacrificed at 15.5 days post-coitum (dpc) by cervical dislocation. The embryos were dissected out, placed in phosphate buffered saline (PBS) solution at 37°C and the umbilical cords cut. The embryos were fixed in 4% paraformaldehyde (PFA) (Electron Microscopy Sciences, Hatfield, PA, USA) overnight and then stored in phosphate buffered solution (PBS) (Life Technologies Inc., Burlington, ON, Canada). Each embryo was individually weighed after transfer to PBS to confirm specimens were of comparable size before treatment. All animal experiments were approved by the animal care committee of the Toronto Centre for Phenogenomics (Toronto, Canada) (Animal Use Protocol: 13-08-120-H). Physical methods of euthanasia were utilized to preserve the form, physiology and functionality of the mouse embryo offspring and performed by trained staff with all efforts made to minimize suffering.

In this study, there were 4 sample preparation protocols. The details of each treatment protocol are described within the appropriate subsections below, however the general descriptions are as follows. Protocol A: samples are immersed in 0.025 N Lugol (Sigma-Aldrich, St Louis, MO, USA). Protocol B: samples are stabilized with hydrogel treatement and immersed in 0.025 N Lugol. Protocol C: samples are immersed in 0.1 N Lugol. Protocol D: samples are stabilized with hydrogel treatment and then immersed in 0.1 N Lugol.

For quantitative analysis, non-stabilized and stabilized mouse embryos were separated into two groups where the optimal treatment protocol was used for each. Group A samples (n = 35) were treated with Protocol A and are fully described in Wong et. al [6] and Group D embryos (n = 4) were treated with protocol D. For visual comparison with Group A, a hydrogel-stabilized embryo was treated with protocol B. To emphasize the stabilization power of the hydrogel matrix in Group D, an embryo without hydrogel pretreatment was treated with a high concentration of Lugol in protocol C. The protocols used for preparation of embryos for visual comparisons were repeated to confirm consistency of results. All samples were scanned using micro-CT after treatment for visual and per-voxel level comparisons.

#### Hydrogel stabilization of embryos

**Figure 2:**
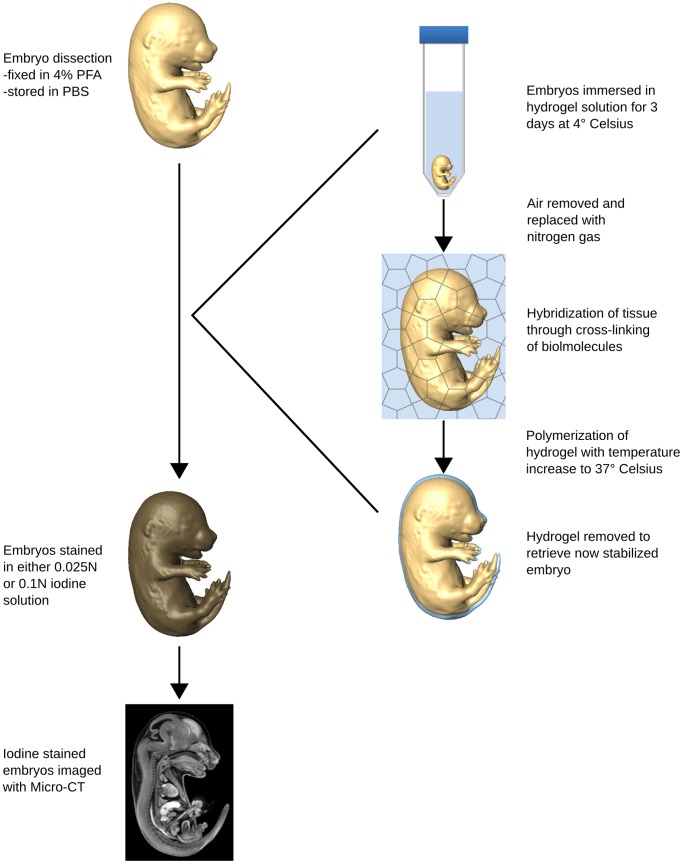
Schematic of the addition of hydrogel stabilization to the mouse embryo micro-CT sample preparation protocol. The stabilization process (right) is added to the standard iodine staining protocol [6] of mouse embryos prior to micro-CT imaging (left).

A previously described tissue preparation protocol for stabilization of mouse brain samples using a hydrogel mesh designated CLARITY was used with some modifications [8]. Briefly, 15.5 dpc mouse embryos were individually immersed in a 20 mL mixture of ice-cold 4% (wt) PFA, 4% (wt/vol) acrylamide (Bio-Rad, Mississauga, ON, Canada), 0.05% (wt/vol) bis-acrylamide (Bio-Rad, Mississauga, ON, Canada), 0.25% VA044 Initiator (Wako Chemicals USA, Inc., Richmond, VA, USA), 0.05% (wt/vol) saponin (Sigma-Aldrich, St Louis, MO, USA) and PBS. To allow for complete tissue diffusion, embryos were left to incubate in the hydrogel solution at 4°C for 3 days. After incubation, the tubes containing the embryo sample were placed in a dessication chamber where any air in the tube was replaced with nitrogen gas. This process is reported to aid hydrogel formation. To initiate the acrylamide polymerization, the mixture was then placed in a 37°C water bath for 3 hours. The samples were then separated from the encasing gel and any extraneous gel was removed. The addition of the hydrogel stabilization step to the existing sample preparation protocol for mouse embryo micro-CT imaging [6] is illustrated in Fig. 2.

#### Iodine staining

The iodine staining protocol was adapted from previously published methods [4], [6], [7], with modification to ensure penetration of the iodine throughout the entire sample. Each sample was immersed in 15 mL stock of either 0.1 N or 0.025 N iodine solution (Sigma-Aldrich, Oakville, Ontario, Canada) for 24 hours on a rotator at room temperature. In the case of samples treated with Protocol C, incubation time was extended to 72 hours to insure penetration of iodine through the hydrogel mesh. All incubations included the replacement of staining solution every 8 hours. At the time of imaging, the samples were embedded in 1% agarose (Bioshop, Burlington, ON, Canada) in 11-mm diameter centrifuge tubes (Beckman Instruments, Palo Alto, CA, USA).

#### Hemotoxylin-eosin (H&E) histological staining

Embryos were paraffin embedded, sectioned in the mid sagittal plane at 5 µm and routinely stained with hematoxylin and eosin. Sections were reviewed by a veterinary pathologist for quality of tissue preservation and staining as well as integrity of cellular and morphological architecture.

### Micro-CT imaging

3D datasets were acquired for each specimen using an SKYSCAN 1172 micro-CT scanner (Billerica, MA, United States). Samples were imaged four at a time using a custom sample holder. Imaging parameters were chosen to optimize image quality for a given 5-hour scan time. Images of embryos stained with 0.025 N Lugol (Protocol A and B) were acquired with the X-ray source at 55 kVp and 181 µA using a 0.5 mm aluminum filter, where each projection was acquired with 12.25 second exposure time. 0.1 N stained (Protocols C and D) embryo images were acquired with the X-ray source at 70 kVp and 142 µA using an aluminum+copper filter, where each projection was acquired with 12.8 second exposure time. All specimens were rotated 360° around the vertical axis, generating 1200 views in less than 5 hours. These views were reconstructed using the Feldkamp algorithm [9] for cone-beam CT. The acquired 3D data block contained 2000×1000×1000 voxel elements of 13.4 µm and was later binned by two to limit the total size of the data for image processing, resulting in a 1000×500×500 3D image with 27 µm isotropic voxels.

### MRI Imaging

To test the effect of hydrogel on embryo magnetic resonance image contrast, a multi-channel 7.0-T MRI scanner (Varian Inc., Palo Alto, CA) with a 6-cm inner bore diameter insert gradient set was used. Prior to imaging, the samples were immersed in 4 mM Gd-DTPA (Magnevist, Bayer HealthCare, Toronto, ON, Canada) for one week. Three custom-built, 14-mm-diameter solenoid coils with a length of 18.3 cm and over wound ends were used to image three mouse embryos in parallel. Parameters used in the scans were optimized for mouse embryo tissue contrast: a 3D gradient echo sequence, with TR/TE = 50/5 ms, 6 averages, 60 degree flip angle, field-of-view 14×14×25 mm and matrix size = 432×432×780 giving an image with 32 µm isotropic voxels [10]. Total imaging time was 15.5 hours.

### Image Registration

The four micro-CT mouse embryos were registered into a consensus, model-independent unbiased average image for both Group A and Group D using an algorithm previously described [6], [11], [12]. The root mean square displacement (RMSD) is calculated from the distance a point in anatomy, in each individual source image, was displaced to reach its equivalent point in the average image. The embryo tails were not included the in RMSD analysis as they are highly variable in position naturally and including them skews the analysis. The population average for each of Group A and D were then registered towards our previously published mouse embryo segmented atlas [6]. The 3D annotations for each of the 48 structures in the 3D atlas were then back propagated to the individual source images through the use of inverse transforms generated from the original image registration. This provides computer-automated calculations of the volumes of the 48 organ structures within each mouse embryo micro-CT image. Normalized volumes are then calculated by determining the percentage each organ structure occupies of the whole mouse embryo volume.

## Results

**Figure 3:**
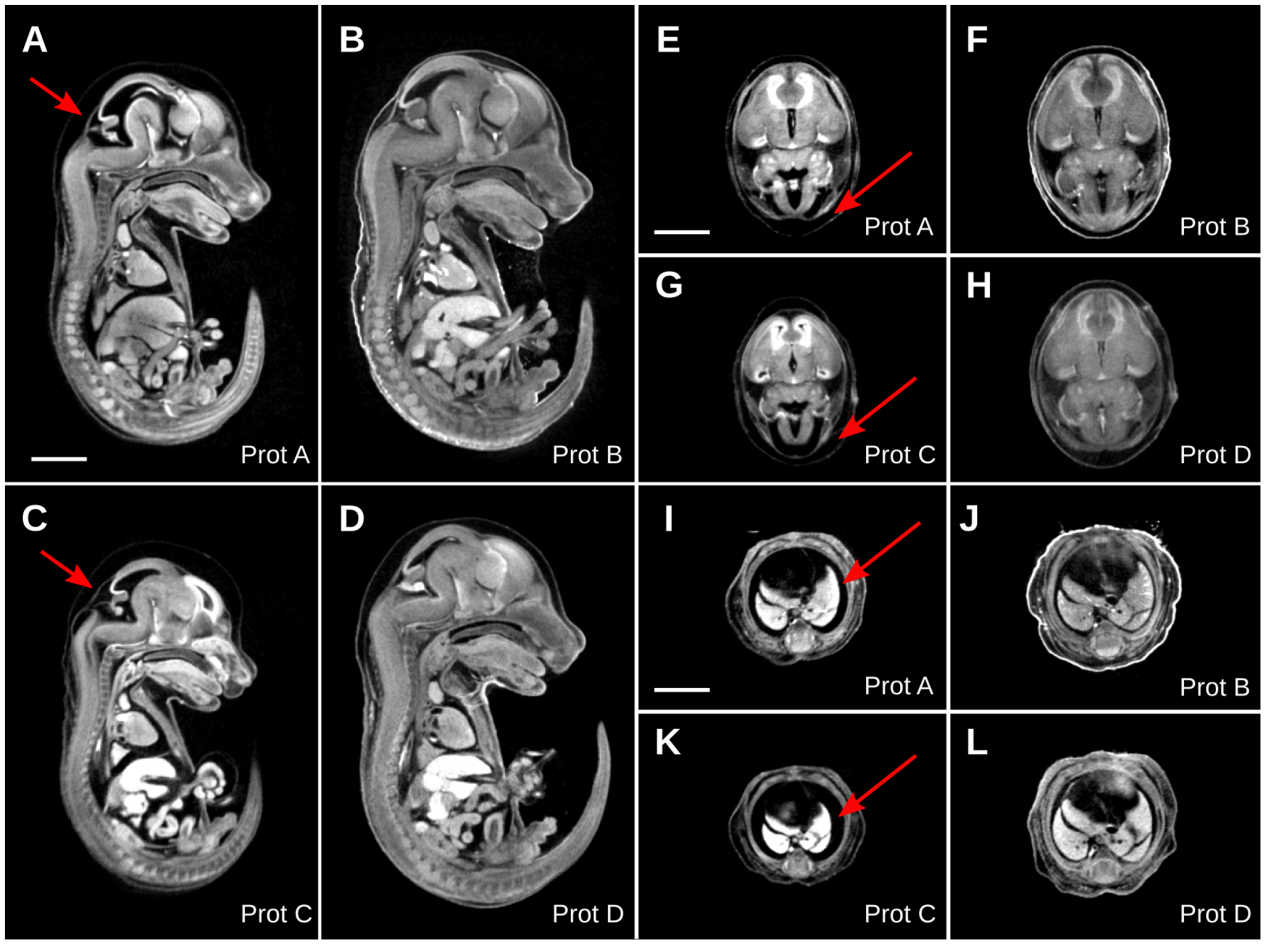
The stabilizing effect of hydrogel pretreatment on iodine-stained whole 15.5 dpc embryos imaged with micro-CT. A, A mid-sagittal micro-CT image of an embryo treated with 0.025 N Lugol (Protocol A). B, An embryo first pre-treated with hydrogel and then immersed in 0.025 N Lugol (Protocol B) C, A mid-sagittal view of an embryo treated with 0.1 N Lugol (Protocol C). D, A hydrogel-stabilized embryo treated with 0.1 N Lugol (Protocol D). Axial brain view of embryo treated with Protocol A (E), Protocol B (F), Protocol C (G), and Protocol D (H). Axial views of thoracic cavities of mouse embryos treated with Protocol A (I), Protocol B (J), Protocol C (K) and Protocol D (L). A–L, Arrows show areas of significant tissue deformation. Scale bar, 2 mm.

To test the efficacy of hydrogel as a stabilizing agent in iodine-stained whole embryo imaging, visual, volumetric and per-voxel level comparisons were made of micro-CT image data. Four treatment protocols were compared. Protocol A, adapted from published methods previously optimized to minimize tissue shrinkage [6], involved a simple immersion soak in 0.025 N Lugol for 24 hours. A mid-sagittal slice through a 15.5 days post coitum (dpc) mouse embryo shows a moderate displacement of the midbrain, neopallial cortex and medulla oblongata brain regions from the outer boundary of the embryo head (Fig. 3A, E). Tissue shrinkage is also evident in the right and left lobes of the lung within the thoracic cavity (Fig. 3I). In contrast, first stabilizing the embryo with a hydrogel mesh (STABILITY) and then treating with 0.025 N Lugol (Protocol B) does not present the same degree of tissue shrinkage of the brain and lungs (Fig. 3B, F, J). Instead, the tissues appear relatively unaffected by the dehydration properties of the iodine treatment and could be readily compared to fixed embryo magnetic resonance imaging (MRI) of specimens, which do not undergo a dehydrating staining process. In addition, the overall volume of the embryo without hydrogel treatment is evidently smaller (20% in volume) (Fig. 3A) than that of the hydrogel stabilized embryo (Fig. 3B) despite a 1% percentage difference in embryo weight before hydrogel stabilization and iodine staining.

In hopes of increasing soft tissue contrast in micro-CT images through increasing the Lugol concentration, another embryo was treated with 0.1 N Lugol (Protocol C − 4x more than currently used) (Fig. 3C, G, K). For comparison, an embryo pretreated with hydrogel and then stained with 0.1 N Lugol (Protocol D) shows markedly less shrinkage and deformation (Fig. 3D, H, L) than its non-stabilized counterpart. Again, before treatment all embryos in this study were of comparable size (<1% difference) yet treatment with a high level of Lugol causes severe tissue shrinkage of the whole embryo (25% in volume) with Protocol C treatment, including brain regions such as the midbrain, neopallial cortex, medulla oblongata, and the lobes of the lung and liver.

Quantitative volume analysis using a mouse embryo 3D segmented atlas [6] reveals that organ volumes measured for embryos treated with protocol A (Group A, n = 35) showed more intersample variance than stabilized mouse embryos treated with protocol D (Group D, n = 12). Specifically, the brain and lung of embryos in Group A were 9.54±0.71% and 1.71±0.20% of whole embryo volumes compared to 10.81±0.58% and 1.70±0.10% for stabilized mouse embryos in Group D. Organ shrinkage and intersample variation due to Lugol can both be greatly reduced by using STABILITY.

**Figure 4:**
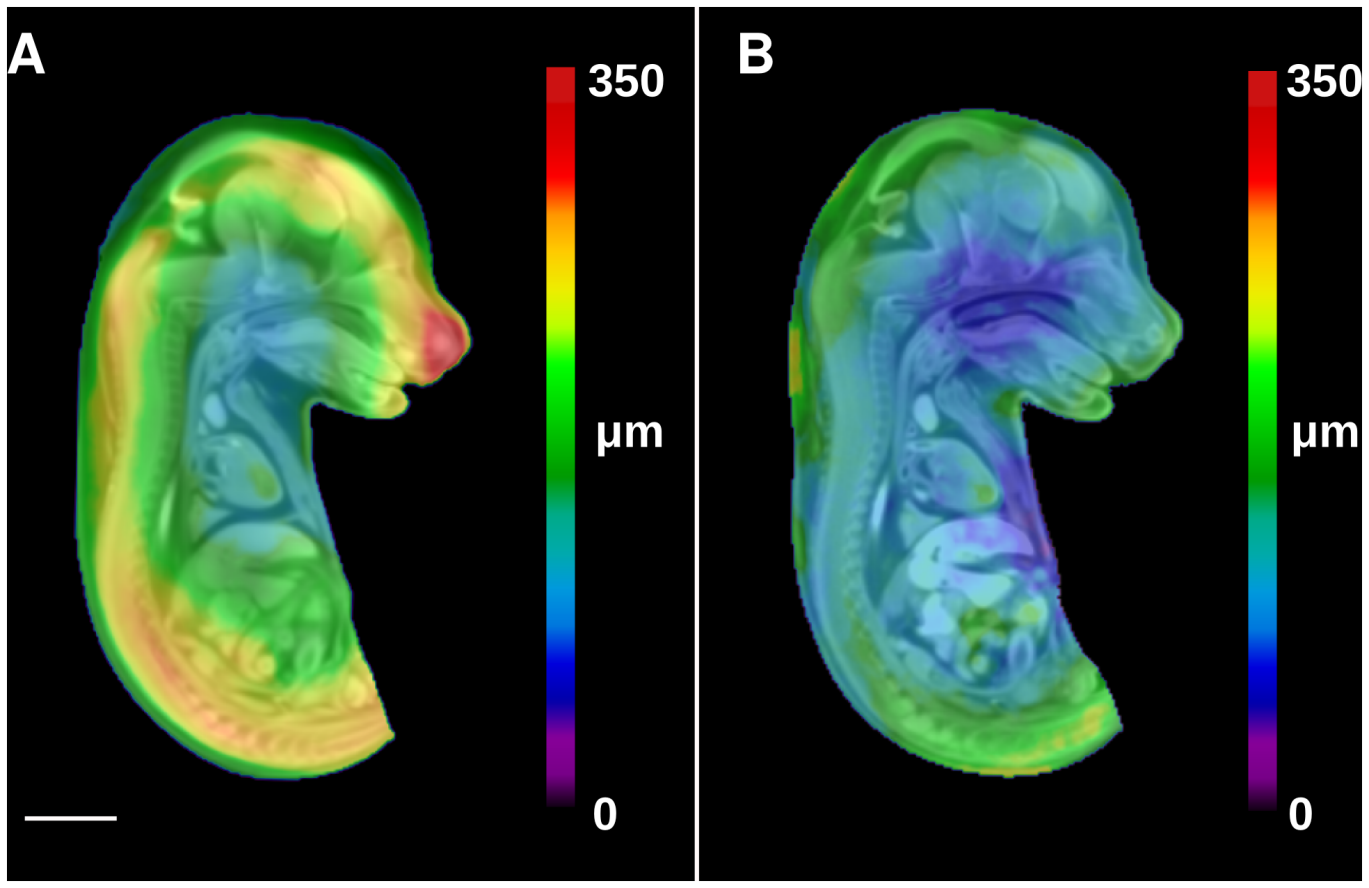
Embryo anatomical variability caused by iodinated contrast agents is reduced with the use of a hydrogel mesh. A, Root mean squared displacement (RMSD) heat map superimposed onto the average embryo image of samples in Group A and treated with Protocol A. B, Group D embryos pretreated with hydrogel with Protocol D show less anatomical variability when analyzed at a per-voxel level. Scale bar, 2 mm.

The effect of STABILITY on the variation in the location of homologous anatomical points is presented in Fig. 4 where the average root mean square displacement (RMSD) to register homologous points is shown as a colour scale. In general, the RMSD at all anatomical locations in the mouse embryo prepared without STABILITY (Fig. 4A) was greater than mouse embryos prepared with the STABILITY protocol (Fig. 4B), especially in the brain. The average RMSD value for mouse embryos in Group A over the whole embryo volume is 222 µm and 227 µm in the brain compared to 171 and 159 µm in Group D using STABILITY.

## Discussion

**Figure 5:**
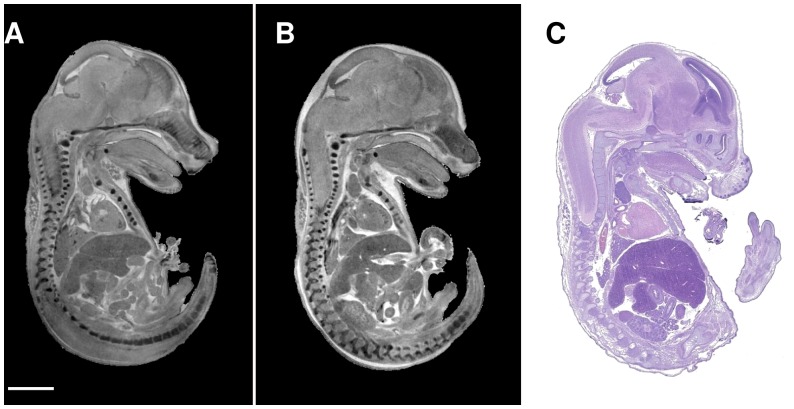
Hydrogel matrix does not effect magnetic resonance image contrast or standard hemotoxylin-eosin (H&E) histological staining. A, Standard T1-weighted magnetic resonance image of a sagittal section of 15.5 dpc embryo anatomy. B, An embryo pretreated with hydrogel shows comparable MRI contrast to A. C, Standard H&E staining of a hydrogel-stabilized embryo immersed in 0.1 N Lugol shows standard quality tissue preservation and staining. Scale bar, 2 mm.

The STABILITY protocol, described here, circumvents the limitations of using iodine staining for embryo micro-CT imaging and allows for the use of higher concentrations of Lugol solution without significant tissue deformation. Removing the obstacle of tissue distortion opens up new possibilities for micro-CT imaging of mouse embryos. The use of STABILITY allows for better X-ray contrast with higher iodine concentrations, shorter scan times and as a result, better overall image quality with increased signal to noise. Since samples stained with Lugol are also subject to additional tissue shrinkage during image acquisition, the use of STABILITY limits shrinkage artifacts, producing sharper images as well. Most importantly, the group variation observed with STABILIZED embryo is tighter, both in terms of normalized organ volumes and anatomic location. This, in turn, will allow for higher sensitivity when using statistical analyses coupled with automated quantitative methods to decipher morphological differences between wild-type and gene-knockout mice [6], [13] In addition, hydrogel pretreatment does not interfere with other imaging modalities in the IMPC primary screening pipeline [3] including standard T1 and T2-weighted MRI (Fig. 5A, B) and hematoxylin and eosin (H&E) histological staining (Fig. 5C). However, diffusion-weighted MRI and diffusion tensor imaging (DTI) of hydrogel-stabilized embryos differ considerably from unstabilized embryos (data not shown).

The method described is neither cost prohibitive nor labor intensive and would be compatible with large-scale phenotyping. Furthermore, the stabilization properties of this method allow for indefinite sample storage before imaging. Finally, the method described is not necessarily limited to the imaging of embryos and could be modified to image other 3D tissue samples.
